# COVID-19 Psychological Impact on Health Care Workers in Saudi Arabia

**DOI:** 10.3390/ijerph18116076

**Published:** 2021-06-04

**Authors:** Hasan S. Alamri, Wesam F. Mousa, Abdullah Algarni, Shehata F. Megahid, Ali Al Bshabshe, Nada N. Alshehri, Awad Alsamghan, Abdullah Alsabaani, Rasha T. Mirdad, Asrar M. Alshahrani, Alya A. Gadah, Almah A. Alshehri

**Affiliations:** 1 Department of Medicine, College of Medicine, King Khalid University, Abha 62527, Saudi Arabia; albshabshe@yahoo.com (A.A.B.); dr_nada@hotmail.co.uk (N.N.A.); 2 College of Medicine, University of Tanta, Tanta 31512, Egypt; wesammousa@hotmail.com; 3 Ministry of Health, Abha 62523, Saudi Arabia; abaid1406@gmail.com (A.A.); almaha.alshehri@hotmail.com (A.A.A.); 4 Department of Family and Community Medicine, College of Medicine, King Khalid University, Abha 61421, Saudi Arabia; shehatafarag@yahoo.com (S.F.M.); asoman@kku.edu.sa (A.A.); dr.alsabaani@hotmail.com (A.A.); 5 Biostatistics Department, High Institute of Public Health, Alexandria University, 65 Garidet St., El Horeya Rd., El Shatby, Alexandria 21526, Egypt; 6 Armed Forces Hospital Southern Region, Khamis Mushait 62413, Saudi Arabia; Rashamirdad1995@hotmail.com; 7 King Fahad Medical City, Riyadh 11564, Saudi Arabia; Asraralshahrani@hotmail.com; 8 King Abdulaziz Medical City, Riyadh 14611, Saudi Arabia; Aliahgd@hotmail.com

**Keywords:** coronavirus, anxiety, depression, observational descriptive study

## Abstract

Objective: Little is known about the impact of Coronavirus (COVID-19) among the health care workers in Saudi Arabia. Therefore, the present study aimed to assess the psychological impact of COVID-19 among the health care workers. Methods: A cross-sectional survey was conducted from May till mid-July among 389 health care workers from government and private hospitals in Saudi Arabia. Data was collected using a pre-structured online questionnaire that measured adverse psychological outcomes, including the Patient Health Questionnaire-9 (PHQ-9) scale and the Generalized Anxiety Disorder 7-item (GAD-7) scale. The Pearson chi-square test was used to assess the distribution of depression and anxiety among health care workers. Results: A high level of anxiety was recorded among the health care workers, and 69.3% of health care workers below the age of 40 were found to have depression. There was a significant increase in depression among staff with chronic health problems (72.1% vs. 61.9%; *p* = 0.048). High anxiety levels were detected among young staff compared to others (68.7% vs. 43.8%; *p* = 0.001). Moreover, 82.1% of the female staff were anxious, as compared to 55.6% of the males (*p* = 0.001). Conclusions: We found increased prevalence of adverse psychological outcomes among the health care workers in Saudi Arabia during the outbreak of COVID-19. Therefore, there is a need for proper screening and development of corresponding preventive measures to decrease the adverse psychological outcomes.

## 1. Introduction

The outbreak of Coronavirus (COVID-19) was declared a pandemic on 1 March 2020 by the World Health Organization [1]. In the following months, quarantine and travel restrictions were imposed in most countries across the world. It is known that there are adverse psychological outcomes associated with an outbreak of infectious disease. The risk of mental diseases like anxiety, post-traumatic stress, depression, and thought disorders increases as the result of containment measures such as self-quarantine and social distancing [2]. Medical workers, in comparison to the public are at a higher risk of experiencing negative psychological effects following a disaster, pandemic situation, or an emergency. Previous studies reported severe emotional stress among the health care workers (HCWs) following the outbreak of infectious diseases [3,4,5]. This includes the severe acute respiratory syndrome (SARS) epidemic in 2003, Ebola virus in 2014, and Middle East Respiratory syndrome (MERS) outbreak in 2015 [6].

There are different factors used to determine the adverse psychological outcomes among the HCWs during infectious disease outbreaks, which are followed with increased mortality rate, uncertain and long quarantine, fear, discrimination, inadequate medical supplies, and stigma [2,7]. Previous studies have shown that the most common predictors of acute post-traumatic stress, chronic post-traumatic stress, and other mental conditions are both an increase in negative coping strategies and, simultaneously, a decrease in support to HCWs [8,9]. There is significant association between the adoption of support HCWs gain from others and their psychological status during the disease outbreak [4]. Another important factor was noted in a study by Bhagavathula et al. [10], which highlighted that the mental health of health workers can be impacted by the lack of knowledge about COVID-19 infection potential and even the widespread misinformation (social media dissemination). The authors discussed the important question of how information is managed, where improving the dissemination of scientific and authentic content can help frontline HCWs in times of public health emergency. There is a significant association between the adoption of support HCWs gain from others and their psychological status during the disease outbreak [4]. Previous studies have shown that the most common predictors of acute post-traumatic stress, chronic post-traumatic stress, and other mental conditions are an increase in the negative coping strategies and a decrease in support to the HCWs [8,9].

The chances of adverse psychological outcomes among HCWs have increased considering the magnitude of COVID-19, especially among the workers performing front line duties. One of the previous studies by Lancee et al. [11] reported a high level of anxiety, post-traumatic stress, emotional stress, and depression among the medical HCWs during, as well as after, the outbreak of infectious diseases. Similarly, a study conducted in Italy revealed that 24.7% of HCWs had depression symptoms, 19.8% had anxiety symptoms, 8.27% had insomnia, 21.9% had perceived stress symptoms, and 49.38% endorsed PTSS [12]. Bai et al. [13] conducted a study in Taiwan and reported that approximately 5% of the HCWs suffered from acute stress disorder. The symptoms of PTSS and acute stress disorder are the same. Therefore, diagnosis of acute stress disorder 3 days to 1 month after trauma is considered a good predictor of PTSS [14]. Ranieri et al.’s [15] findings were obtained in the short and long term after an Italian COVID-19 outbreak. They measured its short-time mental health impact for HCWs, highlighting anxiety as an early reaction for emotional distress and a high risk for post-traumatic stress disorders. The personality dimensions did not mediate the emotional distress as well as probable risk for post-traumatic stress symptoms. The emotional distress was protracted overtime (after 6 months) but in the long term, personality traits were a factor in subjective stress.

There are only few studies that have investigated the psychological impact of the COVID-19 pandemic on the HCWs among the countries that were strongly hit by this infection, like China, United States, India, and Saudi Arabia. Moreover, none of the studies have majorly focused on Saudi Arabian HCWs and assessed the impact of COVID-19 on their psychological well-being. It should be possible to develop and provide effective interventions and treatment to the Saudi population by understanding the psychological outcomes and the related mechanisms among the HCWs during infectious disease outbreaks to improve their psychological well-being. Therefore, the present study aimed to assess the presence of anxiety, depression, and related psychological outcomes among the HCWs following the COVID-19 pandemic. This would help in better understanding the psychological suffering of HCWs and help in developing interventions for alleviating psychological stress among the Saudi population.

## 2. Materials and Methods

### 2.1. Study Design and Sample

A cross sectional survey was conducted, including 389 accessible HCWs during the COVID-19 pandemic in the governmental and private hospitals and primary health care centers in Saudi Arabia.

Tools used to assess mental and psychological health were the Arabic versions of the Patient Health Questionnaire-9 (PHQ-9) scale and the Generalized Anxiety Disorder 7-item (GAD-7) scale [16]. The PHQ-9 is the depression module, which scores each of the nine DSM-IV criteria as “0” (not at all) to “3” (nearly every day) [17]. It has been validated for use in primary care [18] and used to monitor the severity of depression. The total score of PHQ-9 ranges from 0 to 27; scores “0–4” mean no depression, scores “5–9” mean mild depression, scores “10–14” mean moderate depression, scores “15–19” mean moderately severe depression, and scores “20–27” mean severe depression. The GAD-7 is the anxiety module and scores of 5, 10, and 15 are taken as the cut-off points for mild, moderate, and severe anxiety, respectively [19,20].

The data were collected using a pre-structured online questionnaire developed by the researchers after an intensive literature review and expert consultation as a panel of 3 consultants reviewed the items independently. Any discrepancy regarding any item was resolved by consensus at first, then by voting. After agreeing on the final questionnaire format, it was raised online using social media platforms and sent to all accessible staff using a snowball sampling method. The survey remained on the social media platforms from May till mid July 2020. The questionnaire included HCW’s personal data, academic position, qualification, years of experience, chronic health problems, and setting. The amount of contact with COVID-19 cases and history of getting COVID-19 infection were also considered.

Before starting the survey, the participants were informed about the aims of the study and the protection of personal data. They were asked to confirm their informed consent to participate. The study was conducted in accordance with the Declaration of Helsinki, and it was approved by the King Khalid University Research Ethics Committee (Approval Number: ECM#2020-237-HAPO-06-B-001) and the Research Ethical Committee at General Directorate of Health Affairs, Aseer Region, Saudia Arabia.

A pilot study of 20 HCWs was done to assess PHQ-9 and GAD-7 applicability and reliability. Both tools showed α-Cronbach’s of 0.85 and 0.77, respectively.

### 2.2. Data Analysis

All participants’ responses were downloaded and filtered for missing or incorrect data after online submission. After this, the data were extracted, revised, coded, and fed to statistical software IBM SPSS version 22 (SPSS, Inc., Chicago, IL, USA). All statistical analysis was done using two tailed tests. A *p* value of less than 0.05 was statistically significant. Regarding PHQ-9 and GAD-7 scales, all discrete scores were summed and categorized based on the reference section cut off points. Descriptive analysis based on frequency and percent distribution was done for all variables, including demographic data, work data, PHQ-9 and GAD-7 scales. Cross tabulation was used to assess the distribution of HCW’s depression and anxiety status according to their personal and work-related data. Relations were tested using the Pearson chi-square test.

## 3. Results

The study included 389 HCWs from different regions of Saudi Arabia with ages ranging between 20 and 65 years and a mean age of 28.6 ± 10.4 years. The majority of HCWs who completed the questionnaire were males (68.4%) and Saudi (92.8%). In regards to marital status, 69.4% of the responding staff were married and 56.1% had 1–3 children. As for qualification, 39.3% had a bachelor’s degree and 42.7% had post graduate degrees (Master, Fellowship, Board, or PhD). Around 71.5% of the HCWs had no chronic health problems, while allergic or autoimmune problems were reported in 8.7%, diabetes mellitus in 5.9%, and hypertension in 5.4% HCWs (Table 1).

Table 2 demonstrates work data for the HCWs, which shows that 47% of the workers were physicians, 22.1% were technicians, and 17% were nurses. In regards to years of experience, 43.4% had been working for 5–14 years, while 36.5% had worked for less than 5 years. Most HCWs worked in general hospitals (47.8%), followed by primary health care centers (23.7%), and specialist hospitals (19.3%). Around 47.6% of the participants were in direct contact with COVID-19 cases (first line) and only 2.6% had COVID-19 infection.

Regarding PHQ9 items considering depression among the study participants, 73.3% of the HCWs felt tired to different degrees with little energy, followed by little interest or pleasure in doing things (67.6%), trouble falling or staying asleep, or sleeping too much (67.1%). For the global assessment question that checked how difficult these problems made it for the person to work, the answer was 44.5%, 37.8%, 14.4%, and 3.3% for not difficult at all, somewhat difficult, very difficult, and extremely difficult, respectively (Table 3). In total, 35.2% of the HCWs had no detectable depression based on the PHQ9 scale, while mild depression was detected in 27.5%, moderate depression in 18.8%, moderately severe depression in 11.3%, and severe depression in 7.2% of the HCWs (Table 3).

Regarding GAD7 items, Table 4 shows the level of anxiety disorder among the study participants. It shows that 76.1% of the HCWs felt nervous/anxious, 74.8% worried too much about different things, 72% experienced trouble relaxing, 69.9% were easily annoyed or irritable, and 63.3% were not able to stop or control worrying. For the global assessment question that checked how difficult these were for HCWs, the answer was 41.9%, 37.8%, 13.9%, and 6.4% for not difficult at all, somewhat difficult, very difficult and extremely difficult, respectively. In total, 36% of the respondent HCWs had no detectable anxiety, while 32.9% had a mild anxiety level, 13.6% had a moderate anxiety level, and 17.5% had a severe anxiety level.

Table 5 illustrates the distribution of depression and anxiety among health care staff according to their personal data. Depression was detected in 69.3% of HCWs below the age of 40 years as compared to 45.2% of those aged 40 or more (*p* = 0.001). In addition, 79.7% of the female workers had detectable depression in comparison to 57.9% of the males (*p* = 0.001). Depression was significantly higher among staff with chronic health problems (72.1% vs. 61.9%; *p* = 0.048). It was also significantly higher among new staff with less experience than those with high experience (78.2% and 38.1%, respectively; *p* = 0.001). All HCWs who had COVID-19 infection were depressed as compared to 63.9% of others (*p* = 0.018). Anxiety was significantly higher among young staff than others (68.7% vs. 43.8%; *p* = 0.001), and 82.1% of the female staff were anxious, as compared to 55.6% of males (*p* = 0.001).

HCWs who had chronic health problems showed a higher anxiety rate than others (71.2% vs. 61.2%, respectively; *p* = 0.049). Anxiety was also detected in 68.8% of the medical staff compared to 54% of the paramedical group (administrative, technician, and social workers). Moreover, 75.1% of the staff who were in direct contact with COVID-19 cases had detectable anxiety compared to 53.9% of those who were on the second line (*p* = 0.001). Anxiety was detected in 70% of the previously infected staff as compared to 63.9% of those not previously infected.

## 4. Discussion

The aim of this study was to survey the current psychological impact of the Covid-19 pandemic among health care professionals in different regions in Saudi Arabia and discuss the different factors that might have contributed to it. Survey findings revealed that psychological stress exists among health care professionals (*p* = 0.001) and the presence of such stress should be consistently highlighted in the literature to reduce the overflowing anxiety and depression among them, improve patient’s care, and above all afford a healthy working environment. The results of the present study revealed increased prevalence of anxiety and depression among Saudi HCWs during the COVID-19 outbreak. More than half of the HCWs with chronic health problems showed higher anxiety, as compared to others (71.2% vs. 61.2%). These percentages were higher than post-traumatic stress disorder (1–27%) during the SARS outbreak in 2003 [21] and the Ebola outbreak during 2014 and 2016 [7]. The proportion of anxiety and depression reported in this study shows that it was higher in Saudi Arabia, as compared to India and Singapore [22].

The results of the present study show lack of knowledge about psychological coping strategies under these sorts of overwhelming conditions. There might be differences in the psychological health of the workers during infectious disease outbreaks because of the capability and infrastructure of the healthcare system. A similar study was conducted among the Chinese HCWs that reported increased prevalence of anxiety, stress, and depression symptoms [23]. This study also indicated the actual psychological status at the beginning of the COVID-19 outbreak in China [23].

It has been shown that post-traumatic disorder can be manifested through symptoms of hyper-arousal, avoidance, and intrusion after experiencing a traumatic event [24]. Few of the previous studies have confirmed that HCWs develop psychological problems such as stress and depression after a traumatic situation [13,25]. The HCWs performing front line duties during an infectious disease outbreak are also at higher risk of getting infected, especially if they feel any symptoms [13,26]. Moreover, there was also a high risk of transmitting the virus to the families of HCWs because of close contact with them. This made the HCWs more distant and stigmatized. One of the previous studies conducted in China reported three dimensions, fear of infection, perceived high risk of their job, and distancing, that were significantly associated with adverse psychological outcomes [6]. The psychological health of HCWs during an infectious disease outbreak can be improved through friendly mass media and a supportive social environment for alleviating perceived threats among the HCWs. There is also a need to provide HCWs with accurate information and psychological counseling to target stigmatization against the frontline HCWs.

The relationship between people and their environment is improved by focusing on problem solving through active coping strategies, which results in positive emotional responses. In a similar context, the results of the present study have demonstrated that providing sufficient social support and promoting active coping strategies helps in decreasing the development of psychological symptoms such as stress, depression, and anxiety. These results are in agreement with previous studies that showed a buffering effect of social support and active coping strategies on negative psychological health of the HCWs [27]. Relieving the emotional distress is the main aim of passive coping strategies as it is associated with worse psychological health like depression, post-traumatic stress, and anxiety symptoms. It has been shown that it is important to reduce post-traumatic stress among the HCWs through passive coping strategies, so that they are well-aware of the pandemic both mentally and materially.

Low percentages of anxiety and depression in our study and the previous studies might be attributed to the fact that health care professionals experience high levels of satisfaction from their roles caring for others [28] and that religious beliefs have supported the positive effects of spirituality on health care professionals [29]. However, the depressed mood or anxiety exhibited in some healthcare professionals is exacerbated by the extraordinary burden of an augmented work load, inadequate personal protective equipment, the fear of developing COVID-19 virus infection with ensuing infection of friends and relatives, and the need to make ethically tough decisions [30,31].

In this study, it was found that female health care staff and individuals with less experience were more likely to develop symptoms of stress and anxiety, as compared to others. It is expected that the increased level of anxiety among the individuals would be because they would have direct access to patient’s blood or belonged to lower social economic status. These factors are likely to increase the susceptibility of HCWs to COVID-19. No significant differences were detected between nationalities or varying number of children. However, anxiety, but not depression, was significantly higher with a higher number of children, among paramedical staff, and among first line contacts with COVID-19 cases. On the other hand, depression, but not anxiety, was significantly higher among HCWs who were previously diagnosed with COVID-19. Patient care depends heavily on the ability of health care professionals to work in a healthy workplace environment. Thus, we cannot afford to ignore the snowballing anxiety and depression among them [32]. The presence of anxiety/depression among health care professionals as shown in the present study makes the point that the working situation needs to be considered during the COVID-19 pandemic. The recognition of the problem would certainly lead to measures to curb it, although depression/anxiety among workers may be inevitable [33].

The results of the present study indicated more depression and anxiety in those below 40 years of age, females, staff with chronic health problems, new staff with less experience, and previously infected staff. This was similar to the findings reported in frontline medical workers during outbreaks of SARS [21,34]. These findings were also comparable to a meta-analysis of 13 Asian studies that reported a prevalence of 23.2% for anxiety and 22.8% for depression in response to COVID-19 in HCWs [30] and to a more recent meta-analysis [26] that found a similar prevalence of 26% for anxiety and 25% for depression among HCWs.

This study suggests that considering and focusing on the psychological well-being of HCWs in future outbreaks by providing them a supportive workplace is important. Increased attention should be given to the HCWs who are in direct contact with the affected patients [35]. It is important to provide tailored mental health support to the HCWs by considering the amount of stress that they experience. This could be done through the establishment of psychological support groups nationwide, observance of trajectory changes post-pandemic in their mental health condition, and avoiding occurrence of psychiatric disorders. One of the previous studies showed that failure to maintain the psychological well-being of the HCWs would result in increased social and economic burden in the long term [36]. Therefore, there is need to adopt appropriate intervention measures to maintain psychological well-being of the HCWs through timely screening, counselling, creation of a friendly environment, and developing positive coping strategies.

Although the present study has increased knowledge about maintaining psychological well-being of the HCWs, there are several limitations to this study. For instance, the assessment of anxiety symptoms using cut-off values are not accurate as suggested in earlier studies because this is the first study using PHQ-9 and GAD-7 among Saudi HCWs. The study has not performed multivariate analysis, which is a more complicated validation analysis with the inclusion of confirmatory factor analysis (CFA). There is a need to be cautious about making causal relationships without any further follow-up since the cross-sectional study was conducted during the COVID-19 outbreak. However, the present study has provided valuable information about the psychological health of the HCWs in Saudi Arabia during COVID-19, despite these few limitations.

## 5. Conclusions

The study showed increased prevalence of adverse psychological symptoms among the HCWs in Saudi Arabia during the COVID-19 outbreak. Therefore, screening of anxiety and stress among HCWs would help in the identification of post-traumatic stress following the disease outbreak. The main threats experienced by these workers were risk of getting infected, overwhelming workload, stigmatization, and lack of necessary medical supplies. The main risk factors associated with the development of adverse psychological outcomes were maladaptive coping strategies and lack of social support. The negative psychological outcomes could be decreased by preventing early traumatic stress among the HCWs through mitigation strategies and preventive measures. There is also a need for proper screening for adverse psychological outcomes to decrease the negative psychological outcomes of the COVID-19 pandemic among HCWs. Future studies are warranted to scale up to a larger population and to evaluate the effectiveness and feasibility of these interventions.

## Figures and Tables

**Table 1:** Personal data of respondent HCWs, Saudi Arabia, 2020.

Personal Data		No	%
Gender	Male	266	68.4%
	Female	123	31.6%
Age in years	20−	132	33.9%
	30−	184	47.3%
	40−	45	11.6%
	50−	18	4.6%
	60+	10	2.6%
Marital status	Single	109	28.0%
	Married	270	69.4%
	Divorced/widow	10	2.6%
Number of children	None	38	13.6%
	1–3	157	56.1%
	4–5	63	22.5%
	6+	22	7.9%
Qualification	High school	5	1.3%
	Bachelor	153	39.3%
	Diploma	65	16.7%
	Master	33	8.5%
	Board	86	22.1%
	Fellowship	40	10.3%
	PhD	7	1.8%
	None	278	71.5%
Chronic health problems	DM	23	5.9%
	HTN under treatment	21	5.4%
	Mental health problems	16	4.1%
	Cardiac problems	10	2.6%
	Allergic/autoimmune problems	34	8.7%
	Chronic immune deficiency	13	3.3%
	Others	21	5.4%

**Table 2:** Work related data of HCWs, Saudi Arabia, 2020.

Work Related Data		No	%
Job	Social worker	7	1.8%
	Administrative	33	8.5%
	Technician	86	22.1%
	Nursing	66	17.0%
	Dentist	14	3.6%
	Physician	183	47.0%
Experience years	<5 years	142	36.5%
	5–14	169	43.4%
	15–24	57	14.7%
	25+	21	5.4%
Work setting	Primary health care center	92	23.7%
	Specialist hospital	75	19.3%
	University hospital	36	9.3%
	General hospital	186	47.8%
Degree of contact with COVID-19 cases	First line: direct contact with COVID cases	185	47.6%
	Second line: department with no COVID cases	204	52.4%
Diagnosed to have Covid-19 before	Yes	10	2.6%
	No	379	97.4%

**Table 3:** Depression among HCWs in Saudi Arabia during the Covid-19 pandemic.

PHQ9 Items	Not at All	Several Days	More Than Half the Days	Nearly Every Day
	No (%)	No (%)	No (%)	No (%)
Little interest or pleasure in doing things	126 (32.4)	131 (33.7)	64 (16.5)	68 (17.5)
Feeling down, depressed, or hopeless	129 (33.2)	137 (35.2)	64 (16.5)	59 (15.2)
Trouble falling or staying asleep, or sleeping too much	128 (32.9)	123 (31.6)	56 (14.4)	82 (21.1)
Feeling tired or having little energy	104 (26.7)	162 (41.6)	50 (12.9)	73 (18.8)
Poor appetite or overeating	164 (42.2)	109 (28.0)	56 (14.4)	60 (15.4)
Feeling bad about yourself or that you are a failure or have let yourself or your family down	234 (60.2)	92 (23.7)	32 (8.2)	31 (8.0)
Trouble concentrating on things, such as reading the newspaper or watching television	195 (50.1)	101 (26.0)	45 (11.6)	48 (12.3)
Moving or speaking so slowly that other people could have noticed? Or the opposite—being so fidgety or restless that you have been moving around a lot more than usual	222 (57.1)	98 (25.2)	44 (11.3)	25 (6.4)
Thoughts that you would be better off dead or of hurting yourself in some way	337 (86.6)	30 (7.7)	6 (1.5)	16 (4.1)
If you checked off any problems, how difficult have these problems made it for you to do your work, take care of things at home, or get along with other people	Not difficult at all	Somewhat difficult	Very difficult	Extremely difficult
	173 (44.5)	147 (37.8)	56 (14.4)	13 (3.3)
Depression level	Mild depression	Moderate depression	Moderately severe	Severe depression
	107 (27.5)	73 (18.8)	44 (11.3)	28 (7.2)

**Table 4:** Anxiety disorder among HCWs in Saudi Arabia during the Covid-19 pandemic.

GAD7 Items	Not at All	Several Days	More Than Half the Days	Nearly Every Day
	No (%)	No (%)	No (%)	No (%)
Feeling nervous, anxious or on edge	93 (23.9)	167 (42.9)	76 (19.5)	53 (13.6)
Not being able to stop or control worrying	143 (36.8)	131 (33.7)	52 (13.4)	63 (16.2)
Worrying too much about different things	98 (25.2)	155 (39.8)	70 (18.0)	66 (17.0)
Trouble relaxing	109 (28.0)	142 (36.5)	77 (19.8)	61 (15.7)
Being so restless that it is hard to sit still	196 (50.4)	105 (27.0)	46 (11.8)	42 (10.8)
Becoming easily annoyed or irritable	117 (30.1)	143 (36.8)	62 (15.9)	67 (17.2)
Feeling afraid as if something awful might happen	179 (46.0)	116 (29.8)	39 (10.0)	55 (14.1)
If you checked off any problems, how difficult have these problems made it for you to do your work, take care of things at home, or get along with other people	Not difficult at all	Somewhat difficult	Very difficult	Extremely difficult
	163 (41.9)	147 (37.8)	54 (13.9)	25 (6.4)
Anxiety level	Mild depression	Moderate depression	Moderately severe	Severe depression
	140 (36.0)	128 (32.9)	53 (13.6)	68 (17.5)

**Table 5:** Distribution of depression and anxiety among HCWs according to their personal data.

Personal Data		Depressed	Anxious
		No (%)	No (%)
Age in years	<40 years	219 (69.3)	217 (68.7)
	>40 years	33 (45.2)	32 (43.8)
*p*-value		0.001 *	0.001 *
Gender	Male	154 (57.9)	148 (55.6)
	Female	98 (79.7)	101 (82.1)
*p*-value		0.001 *	0.001 *
Number of children	None	26 (68.4)	20 (52.6)
	1–3	100 (63.7)	99 (63.1)
	4–5	30 (47.6)	29 (46.0)
	6+	13 (59.1)	9 (40.9)
*p*-value		0.109	0.048 *
Chronic health problems	No	172 (61.9)	170 (61.2)
	Yes	80 (72.1)	79 (71.2)
*p*-value		0.048 *	0.049 *
Job	Paramedical	78 (61.9)	68 (54.0)
	Medical	174 (66.2)	181 (68.8)
*p*-value		0.411	0.004 *
Experience years	<5 years	111 (78.2)	109 (76.8)
	5–14	102 (60.4)	104 (61.5)
	15–24	31 (54.4)	26 (45.6)
	25+	8 (38.1)	10 (47.6)
*p*-value		0.001 *	0.001 *
Degree of contact with COVID-19 cases	First line: direct contact with COVID cases	126 (68.1)	139 (75.1)
	Second line: department with no COVID cases	126 (61.8)	110 (53.9)
*p*-value		0.191	0.001 *
Diagnosed to have Covid-19 before	Yes	10 (100.0)	7 (70.0)
	No	242 (63.9)	242 (63.9)
*p*-value		0.018 *	0.689

*p*: Pearson X2 test; * *p* < 0.05 (significant).

## Data Availability

The data set used and analyzed during the study is available from the corresponding author upon reasonable request.
